# Anomalies in the Geometric Surface Structure of Shaped Elements Composed of Inconel 718 Alloy

**DOI:** 10.3390/ma14247524

**Published:** 2021-12-08

**Authors:** Bartłomiej Krawczyk, Piotr Szablewski, Stanisław Legutko, Krzysztof Smak, Bartosz Gapiński

**Affiliations:** 1 Faculty of Mechanical Engineering, Poznan University of Technology, 3 Piotrowo Street, 60-965 Poznan, Poland; stanislaw.legutko@put.poznan.pl (S.L.); bartosz.gapinski@put.poznan.pl (B.G.); 2 Pratt & Whitney Kalisz, 4a Elektryczna Street, 62-800 Kalisz, Poland; piotr.szablewski@prattwhitney.com (P.S.); krzysztof.smak@prattwhitney.com (K.S.); 3 Higher Vocational State School President Stanislaw Wojciechowski in Kalisz, 4 Nowy Świat Street, 62-800 Kalisz, Poland

**Keywords:** Inconel 718, turning, surface roughness, microstructure

## Abstract

This paper presents the results of investigation that was performed on shafts composed of Inconel 718. Tests were performed in dry and wet conditions. Cutting parameters, such as feed and depth of cut, were constant. The cutting speed was changed. The investigation was performed for various shaft shapes: cylindrical, taper 30°, taper 45°, and sphere. For that reason, the value of the angle between the machined surface and the cutting edge changed. The lowest values of the roughness parameters, *Ra* and *Rz*, were obtained for a larger value of the angle between the machined surface and cutting edge. The investigation showed that cutting speed, machining conditions (dry and wet machining), and the variable angle between the machined surface and the cutting edge influenced the surface roughness. Application of a higher cutting speed resulted in lower roughness values. Lower values of roughness parameters were obtained by wet machining.

## 1. Introduction

Inconel 718 belongs to the group of heat-resistant alloys based on nickel, also called superalloys [1] or high-strength alloys [2]. Due to its mechanical and physical properties, such as high strength [3] and creep resistance at high temperatures, it is widely used in the aircraft and aerospace industries, as well as in gas turbines and nuclear reactors [4,5,6,7]. The numerous advantages of this alloy result in it being used in extraordinarily severe conditions, such as those inside the combustion chamber of an aircraft engine [8,9], where heat resistance [10,11] and precision of the manufacturing of parts are extremely important, because they influence the safety of passengers during flight. Due to the price of Inconel and machining difficulties [12,13], it is used only where conditions require it. Unfortunately, the uniqueness of the alloy means that very few descriptions of it can be found in literature.

One of the challenges during the machining of Inconel is ensuring the required surface finish [14]. In the case of turning, one can find a few different cutting parameters that influence the roughness change [15,16,17]. Its evaluation can be performed by conducting a test and credibly assessing the roughness parameters [18]. Coelho et al. [19] investigated the influence of the shape and material of the cutting insert on the surface roughness. In those tests, inserts made of ceramics (coated and uncoated) and PCBN with standard and modified geometry were used. The modification of the insert did not unequivocally affect the surface roughness. Depending on the shape of insert, the *Ra* parameter increased or decreased. The influence of the cutting tool material on the roughness also was investigated by Ezugwu and Tang [20]. In those tests, ceramic inserts with two types of coatings, ZrO_2_ and TiC, were used. The results of that work showed that machining with mixed oxide ceramics (Al_2_O_3_ + TiC) produced better surface roughness, and the same was observed with a round insert instead of a rhomboid-shaped one. Yang Hua and Zhanqiang Liu performed a dry turning test [21]. The researchers showed that changing the cutting speed had no significant influence on the surface roughness. In the same paper, the authors proved that a larger cutting edge radius was much more effective than a smaller one, with regard to the improvement of the surface quality. A similar relationship was proved when the feed rate was decreased. The effect of cutting speed on roughness was investigated by D’Addon et al. [22]. They conducted a study in which turning of Inconel 718 was performed at various cutting speeds. It was found that the longer the contact of the cutting edge with the material, the worse the quality of the surface was. However, it was noticed that the differences were not significant when comparing the first and third passes. The scientists observed that an increase in the cutting speed resulted in an improvement of the roughness, but for certain values, the effect was the opposite. This could be due to the fact that the cutting edge became worn rapidly. Similar conclusions concerning the cutting parameters’ influence on the surface quality were drawn by Jafarian and Umbrello [23]. In their study, the optimum values of the cutting parameters for roughness and tool life were determined using an intelligent neural network. The influence of coolant on turning quality was investigated by Kumar et al. [24]. They proved the well-known assumption that a lack of cooling causes the worst surface quality of the nickel-based superalloy. Using the carbide tool, the superiority of the oil mist cooling method known as minimum quantity lubrication (MQL) over the traditional method of cooling also was proved. Machining in the wet state and with MQL, in addition to reduction of the temperature in the cutting zone, provided the desired chip brittleness, which tended to improve the quality of the treated surface. It also helped to reduce the contact of the tool with the chips, and consequently, the tool life was extended. An analogical study was presented by Maruda et al. [25]. Slightly broader studies were undertaken by Mehta, Hemakumar et al. by performing experiments with various combinations of coolant, such as MQ, cryogenic, and cold air [26]. It was reported that the best surface quality was obtained with the combination of MQL and cryogenic, for which the results were approximately 85% better compared to dry turning. Zhenlong Pend and colleagues compared conventional turning to high-speed ultrasonic vibration cutting (HUVC) [27,28]. The investigation proved that HUVC-aided machining had a positive effect on the *Ra*, *Rz,* and *Rt* values. Those benefits were much greater with increasing cutting edge wear.

The present work shows the results of experimental turning of variously shaped workpieces. The influences of the coolant, cutting speed, hardness, and shape of the machined material on the *Rz* and *Ra* parameters were assessed.

## 2. Materials and Methods

Our investigations were performed on shafts with an external diameter of 50 mm and a length of 80 mm (Carpenter Technology, Philadelphia, PA, USA). The samples were composed of Inconel 718 (Carpenter Technology, Philadelphia, PA, USA) with a hardness of 23 ± 2 HRC and 36 ± 2 HRC (Carpenter Technology, Philadelphia, PA, USA). Negative cutting inserts, WNMG 080408-MS KC5510 with approach angle *k_r_* = 95° (Kennametal, Tianjin, China), and tool shank DWLNL 2525 M08 (Sandvik Coromant, Gimo, Sweden) were applied. The cemented carbide inserts (Kennametal, Tianjin, China). used in the investigation had a corner radius of 0.8 mm, and were coated in a PVD process. Cutting parameters were selected based on previous tests: *v_c_* = 45–70 m/min; *f* = 0.2 mm/rev; *a_p_* = 1 mm.

**Figure 1:**
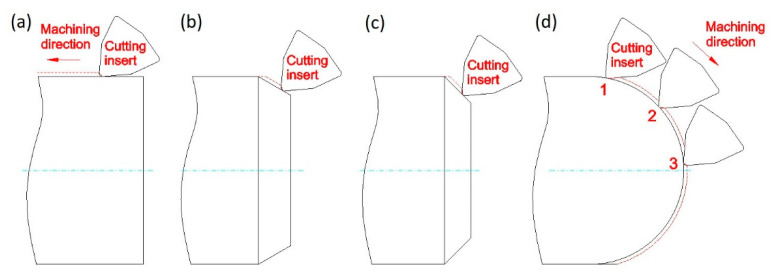
The scheme of machining direction for: (**a**) longitudinal turning; (**b**) taper turning 30°; (**c**) taper turning 45°; (**d**) sphere turning.

**Figure 2:**
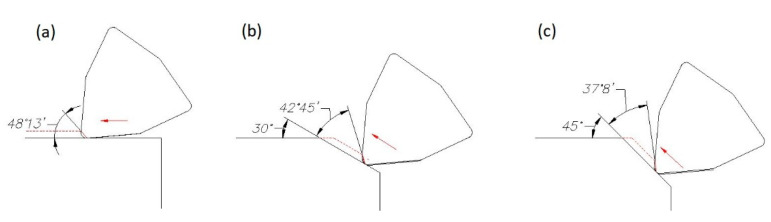
Change of the angle between the main cutting edge and the machined surface during: (**a**) longitudinal turning; (**b**) taper turning 30°; (**c**) taper turning 45°.

The investigation was performed with the use of longitudinal turning, transverse turning, taper (Famot, Pleszew, Poland) turning at angles of 30° and 45°, and sphere turning. A diagram of the machining execution can be seen in Figure 1. According to Colwell [29], an angle between the main cutting edge and the machined surface was graphically determined, and is shown in Figure 2. The angle was contained between the machined surface of the sample and a straight line passing through the two points defined on the cutting edge. The first point was the one on the secondary cutting edge that was still in the contact with machined surface. The second point was located on the cutting edge, and was determined by the cutting depth. 

**Figure 3:**
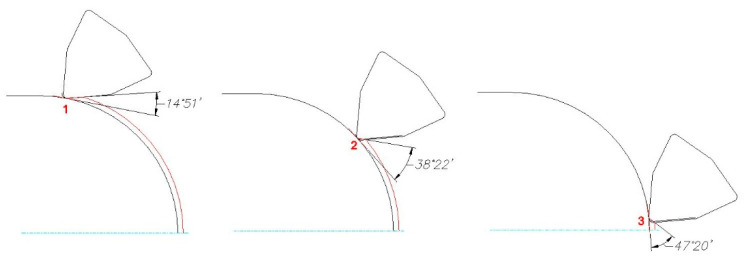
Change of the angle between the main cutting edge and the machined surface during sphere turning.

The surface roughness on the sphere was measured in three locations (Figure 3).

The turning tests were performed on a CNC lathe in dry and wet conditions. Ecocool Global 10 lubricant (Fuchs, Hanley, UK), produced by Fuchs Oil Corporation, was used in the tests. It is an 8% emulsion concentrate based on mineral oil and is 92% water. This lubricant is intended for the aircraft industry.

The surface roughness was measured with a Hommel Tester T1000 profilometer (Jenoptik, Villingen Schwenningen, Germany). The roughness parameters were measured using *l_r_* = 0.8 mm and *l_n_* = 4.8 mm. Photographs of the microstructures were taken with the use of an EPIPHOT 200 microscope (Nikon, Tokyo, Japan) made by Nikon. The samples were mounted in duracryl (Buehler, Lake Bluff, IL, USA) and etched with Kallings reagent (Chmes, Poznan, Poland) after polishing.

Depending on the shape of the machined surface, the angle between the cutting edge and the machined surface changed. This meant that the length of the active edge in the material also changed. In order to show the influence of the process dynamics on the surface roughness, the Δ*Rz* coefficient was calculated:

(1) $$\Delta Rz=\frac{Rz_{i}}{Rz_{to}}$$

where:

*Rz_i_*—measured height of surface roughness;
*Rz_to_*—theoretical surface roughness, which depends on the minimum uncut chip thickness value, condition of the cutting edge, and the dynamics of the machining process.

The *R* profile of the roughness can be defined as:

*Ri_to_* = *Ri_o_* + ∆*Ri_h_* + ∆*Ri_d_* (2)

where:

*R_io_*—foreseen (theoretical) component of the machined surface roughness, *R_i_*, as a result of the so-called efficient kinematic-geometrical projection of the cutting insert in the material;
Δ*R_ih_*—a component of the *R_i_* parameter of roughness that is an effect of the minimum uncut chip thickness value *h_min_* [30,31];
Δ*R_id_*—component of the *R_i_* parameter resulting from the dynamic and tribological effects (vibrations) in the zone of the cutting insert’s contact with the material, varying during machining in a permanent, periodical, or random mode.

In the conditions of the so-called arc projection of the cutting insert in the material [32,33], the first two components of Formula (2), referring to the *Rz* parameter, are described by the relationships:

(3) $$Ri_{o}=\frac{f^{2}}{8\;r_{\varepsilon }}$$

(4) $$\Delta Ri_{h}=\frac{k\;\cdot \;r_{n}}{2}\;\left(1+\frac{k\;\cdot \;r_{n}\;\cdot r_{\varepsilon }}{f^{2}}\right)$$

and

(5) $$Rz_{to}=\;\frac{f^{2}}{8\;r_{\varepsilon }}+\;\frac{k\;\cdot \;r_{n}}{2}\;\left(1+\;\frac{k\;\cdot \;r_{n}\;\cdot \;r_{\varepsilon }}{f^{2}}\right)+\;\Delta Rz_{d}$$

where:

*f*—feed;
*r_n_*—cutting edge radius;
*r_ε_*—nose radius;
*k*—(*h_min_/r_n_*); factor (*k* ≈ 0.1 ÷ 0.6).

Based on previous research, in calculations, the cutting edge radius was assumed as *r_n_* = 0.015 mm (approximately). By transforming Formulas (2)–(5) into the form:

(6) $$Rz/Rz_{to}=1+\;\frac{\frac{k\;\cdot \;r_{n}}{2}\;\left(1+\;\frac{k\;\cdot \;r_{n}\;\cdot \;r_{\varepsilon }}{f^{2}}\right)+\;\Delta Rz_{d}}{Rz_{to}}=1+A$$

it can be seen that the level of anomalies (deviations) of the roughness parameter *Rz* from *Rz_to_* is determined by the component *A,* depending on the factors related to macro- and microgeometry of the cutting insert (*r_ε_*, *r_n_*), factor *k*, feed, and the characteristics of the contact zone of the cutting edge with the machined material.

## 3. Results and Discussion

**Figure 4:**
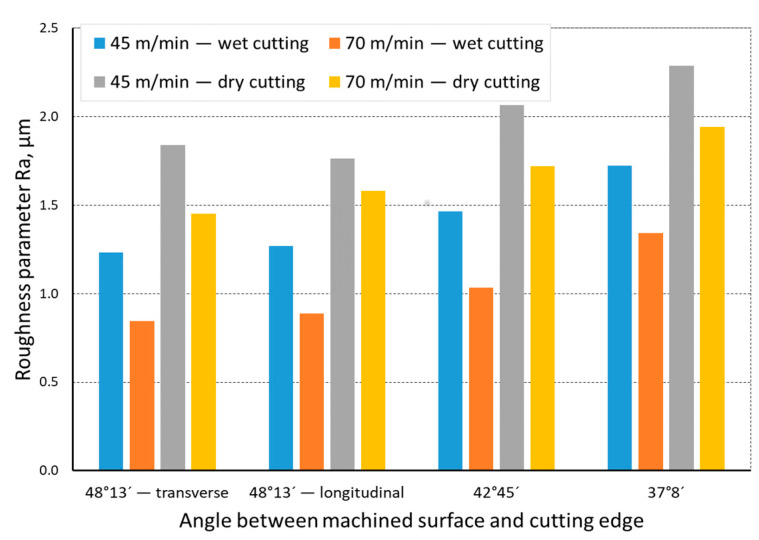
The impact of cutting speed on the variability of roughness parameter *Ra* in the function of the angle between the machined surface and cutting edge for a rectilinear track and a material hardness of 23 HRC.

**Figure 5:**
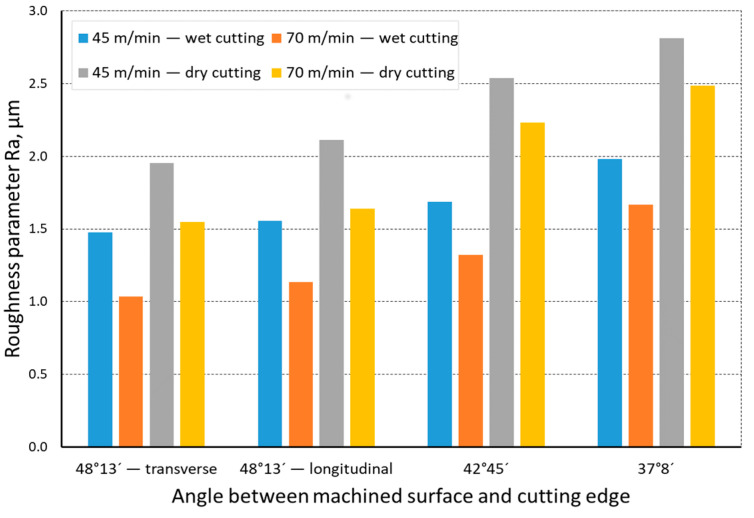
The impact of cutting speed on the variability of roughness parameter *Ra* in the function of the angle between the machined surface and cutting edge for a rectilinear track and a material hardness of 36 HRC.

**Figure 6:**
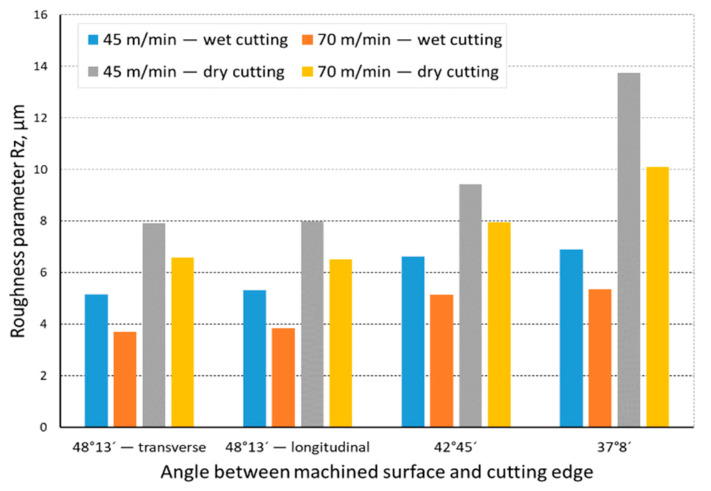
The impact of cutting speed on the variability of roughness parameter *Rz* in the function of the angle between the machined surface and cutting edge for a rectilinear track and a material hardness of 23 HRC.

**Figure 7:**
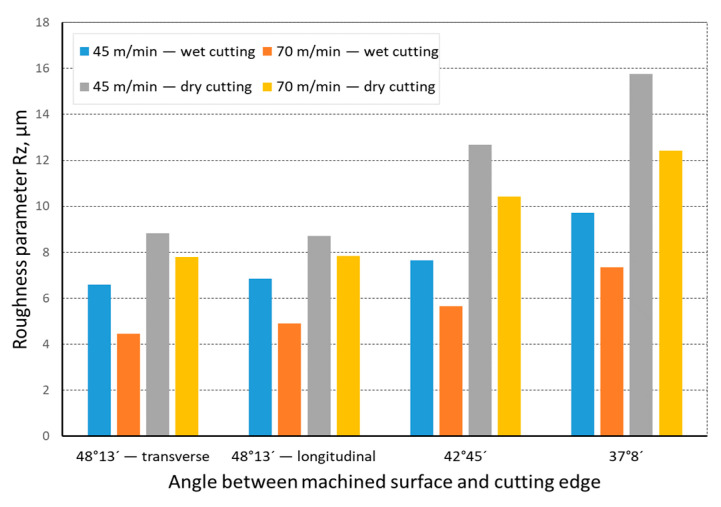
The impact of cutting speed on the variability of roughness parameter *Rz* in the function of the angle between the machined surface and cutting edge for a rectilinear track and a material hardness of 36 HRC.

The quality of the machined surface was assessed depending on the cutting speed, conditions of machining (dry machining and machining with cooling), various hardness levels of the machined material (before and after heat treatment), and the shape of the part being processed (and consequently, the variable angle between the cutting edge and the machined surface). The quality of the surface was assessed with the application of the *Ra* and *Rz* parameters. In an analysis of the influence of the cutting speed on the surface quality, it was found that the *Ra* and *Rz* parameters improved at a higher cutting speed; i.e., for *v_c_* = 70 m/min. For the higher cutting speed, a better surface quality was obtained regardless of the shape under machining. The value of the assessed *Ra* parameter for straight-line motion, both for material prior to heat treatment (Figure 4) and after heat treatment (Figure 5), improved by 30%, particularly for longitudinal and transverse turning, when the angle between the machined surface and the cutting edge was 51°51′. Reduction of that angle when turning taper surfaces with the angles of 30° and 45° resulted in the improvement of the surface quality assessed by the *Ra* parameter dropping below 30%, but it was still visible. The recorded *Rz* parameter also reached lower values when a higher cutting speed (*v_c_* = 70 m/min) was applied, regardless of the machined shape (Figure 6 and Figure 7). For all the cases under investigation, improvement of the *Rz* parameter was at least 20%. In the tests of material before and after heat treatment was used, the hardness of the samples was 23 HRC and 36 HRC, respectively. Based on the obtained results, it was seen that an increase in hardness negatively influenced the roughness parameters, both *Ra* and *Rz*.

The increase in the *Ra* parameter was above 15% regardless of the shape under examination; the *Rz* parameter increased above 25%. It would be recommendable, therefore, to machine parts of that material in the “soft” condition (before heat treatment); however, this is not always possible. One of the reasons is that the material is supplied by the producers in an already-hardened condition. Another reason is deformation of the part during heat treatment due to the residual stresses generated during machining of the Inconel 718 material. Machining in the soft condition can, therefore, be applied for elements with wide fields of tolerance and admissible significant errors of shape and position. Arunachalam et al. [34] showed that when machining Inconel 718 with a CBN cutting insert, compressive stresses were obtained on the machined surface; whereas Salio et al. [35] showed in their work that as a result of turning this material with carbide cutting inserts at a depth of x < 20 µm, tensile stresses arose, and compressive stresses arose at a depth of x = 25–50 µm (from the machined surface). Pawade et al. [36] noted during their research an increase in tensile stress with increasing feed; this increase was also recorded when reducing the depth of cut. Sharman et al. [37] used cemented carbide inserts with TiN/Al_2_O_3_/TiN antiwear coating and without coating in their research. The use of coated cutting inserts resulted in stress that was two times larger. This fact may be related to the cutting edge radius *r_n_*, which is larger in the case of coated inserts.

**Figure 8:**
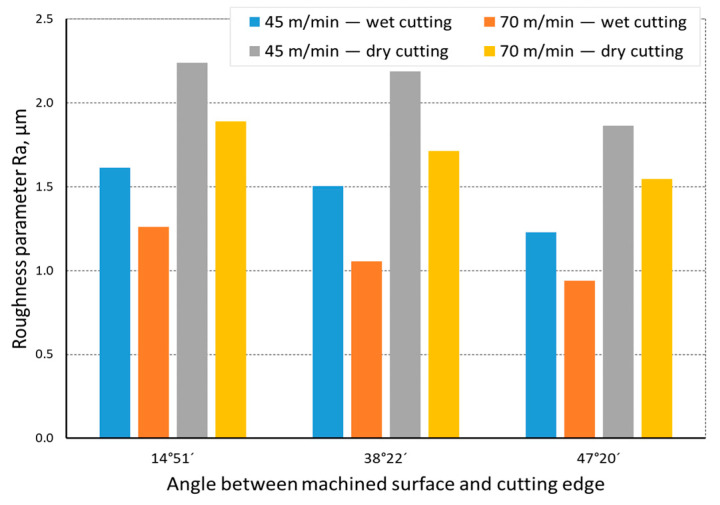
The impact of cutting speed on the variability of roughness parameter *Ra* in the function of the angle between the machined surface and cutting edge for a curvilinear track and a material hardness of 23 HRC.

**Figure 9:**
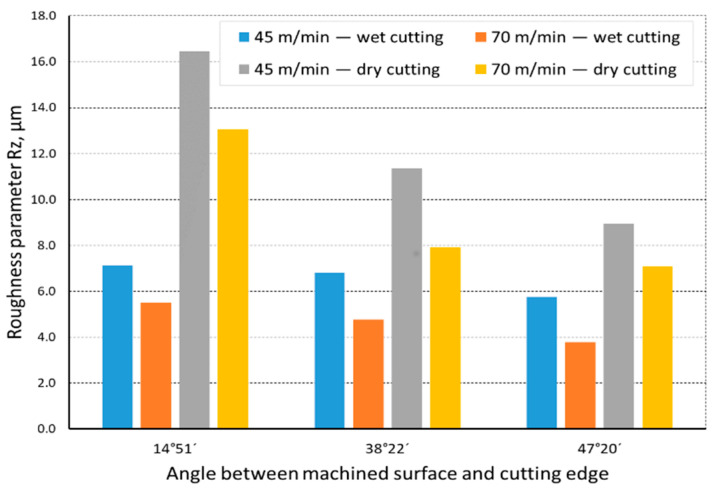
The impact of cutting speed on the variability of roughness parameter *Rz* in the function of the angle between the machined surface and cutting edge for a curvilinear track and a material hardness of 23 HRC.

**Figure 10:**
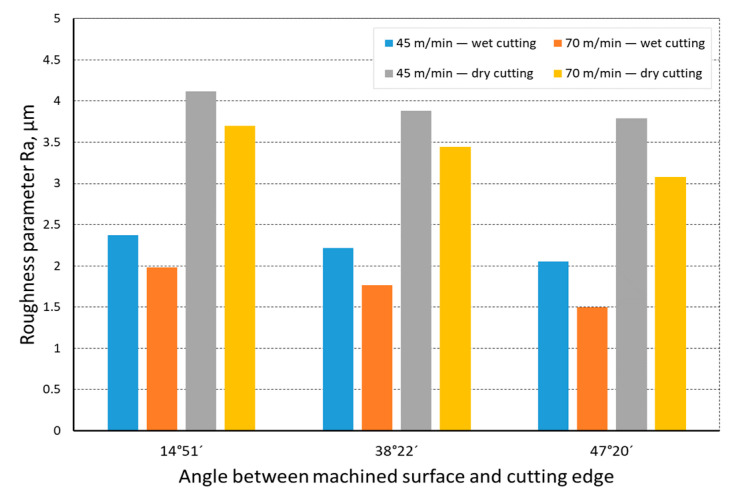
The impact of cutting speed on the variability of roughness parameter *Ra* in the function of the angle between the machined surface and cutting edge for a curvilinear track and a material hardness of 36 HRC.

**Figure 11:**
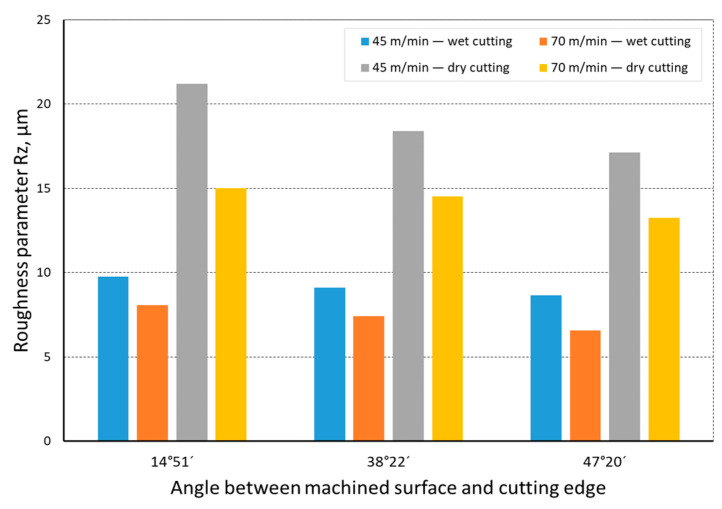
The impact of cutting speed on the variability of roughness parameter *Rz* in the function of the angle between the machined surface and cutting edge for a curvilinear track and a material hardness of 36 HRC.

When analyzing the influence of the machined part shape, and consequently, the variable angle between the cutting edge and the machined surface, it was found that a reduction in that angle deteriorated the quality of the machined surface (Figure 4, Figure 5, Figure 6, Figure 7, Figure 8, Figure 9, Figure 10 and Figure 11). Reduction in the angle between the machined surface and the cutting edge influenced the length of the active cutting edge, which grew. A longer active cutting edge caused an increase in resistance during machining, which resulted in deterioration of the machined surface quality. In turning with a linear trajectory of the cutting edge motion, the angle between the cutting edge and the machined surface was constant; in turning with a curved trajectory, the value of that angle was variable, depending on the position of the edge in relation to the element under machining. The angle increased with the relocation of the edge on a spherical element. It should be pointed out that during turning of a spherical surface, the cutting edge, at a certain moment, reached a position in which the value of the angle between the cutting edge and the machined surface was close to the value of the angle that occurred in turning the tapered surface with an angle of 45°.The values of those angles were 45°–39°52′ for the tapered surface and 40°50′ (place of measurement no. 2). As mentioned above, in machining a tapered surface, the angle was constant; the stated angle value of 40°50′ should be taken as an approximate one. Nevertheless, it could be expected that the compared roughness parameters, *Ra* and *Rz,* would have values close to each other. However, after machining the material of 23 HRC hardness, at both considered cutting speeds and regardless of the machining conditions, the recorded *Ra* parameter for a spherical surface was over 10% lower than that for the tapered surface. A different situation was observed for the material with 36 HRC hardness. The values of the *Ra* parameter for machining with cooling for spherical and tapered surfaces were close to each other. On the other hand, in dry machining, the value of the *Ra* parameter for the spherical surface was significantly higher. In the case of cutting speed, *v_c_* = 45 m/min, the average value was *Ra* = 2.814 µm for the tapered surface; for the spherical surface, the value was *Ra* = 3.880 µm. Therefore, the increase in that parameter was 37%.

**Figure 12:**
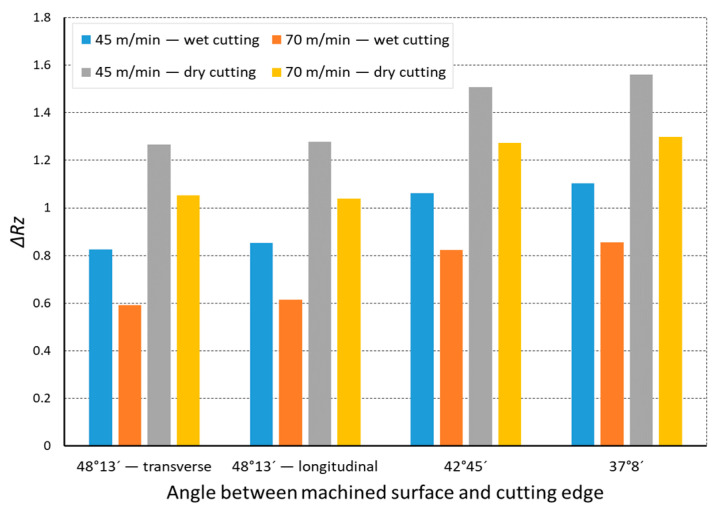
The impact of angle between the machined surface and cutting edge on ratio Δ*Rz* of the average measured surface roughness height to the theoretical surface roughness *Rz_to_* for a rectilinear track and 23 HRC.

**Figure 13:**
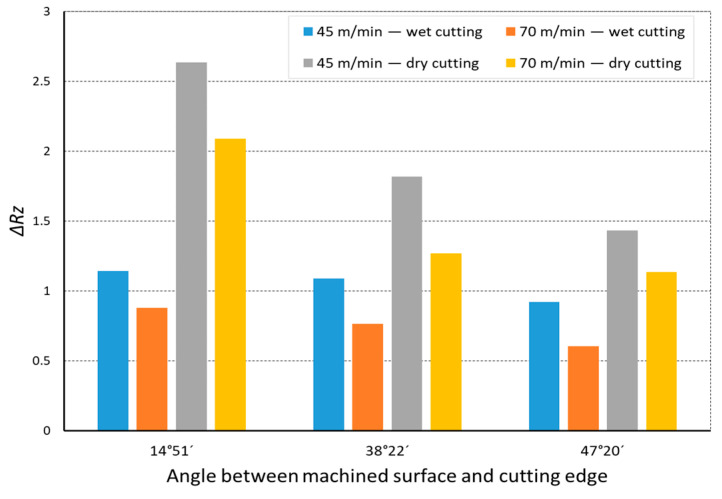
The impact of angle between the machined surface and cutting edge on ratio Δ*Rz* of the average measured surface roughness height to the theoretical surface roughness *Rz_to_* for a curvilinear track and 23 HRC.

**Figure 14:**
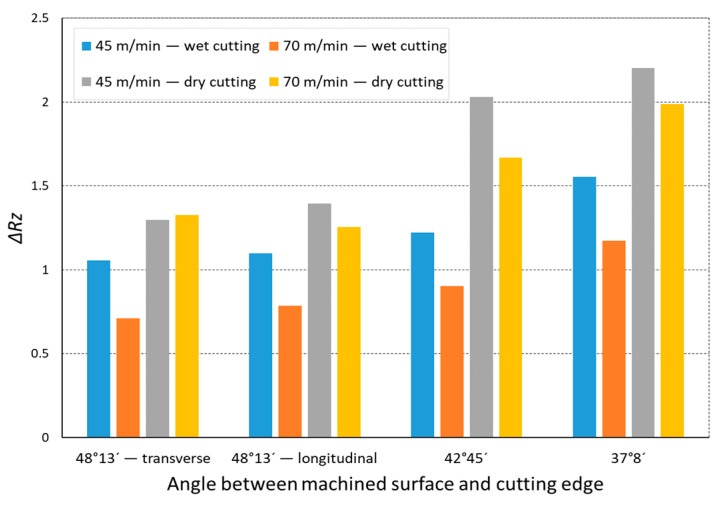
The impact of angle between the machined surface and cutting edge on ratio Δ*Rz* of the average measured surface roughness height to the theoretical surface roughness *Rz_to_* for a rectilinear track and 36 HRC.

**Figure 15:**
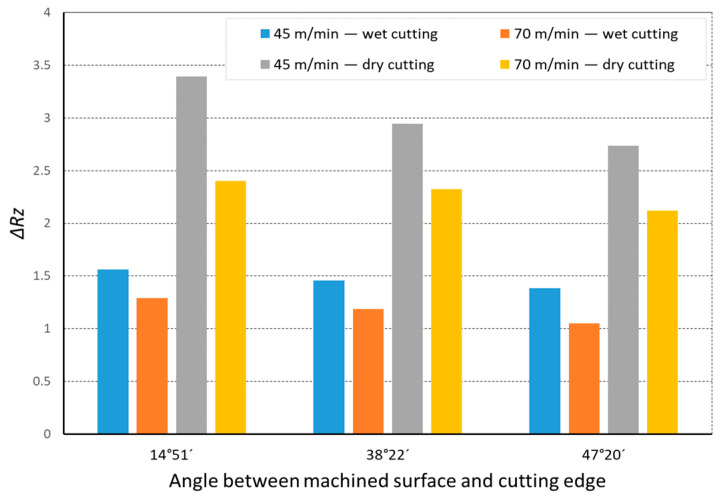
The impact of angle between the machined surface and cutting edge on ratio Δ*Rz* of the average measured surface roughness height to the theoretical surface roughness *Rz_to_* for a curvilinear track and 36 HRC.

**Figure 16:**
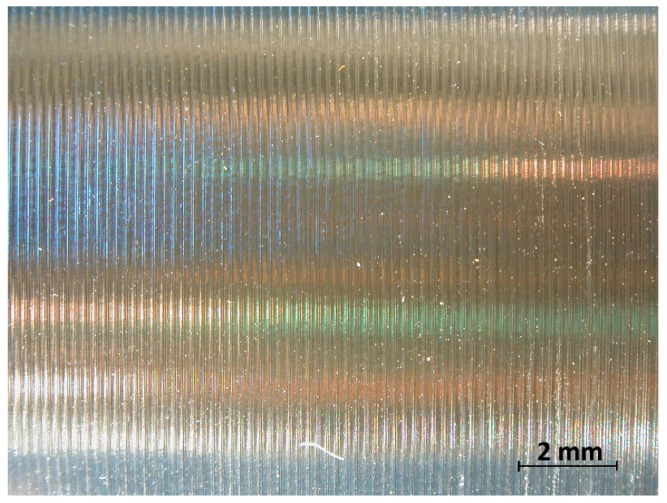
View of the machined surface after longitudinal turning without coolant (36 HRC).

**Figure 17:**
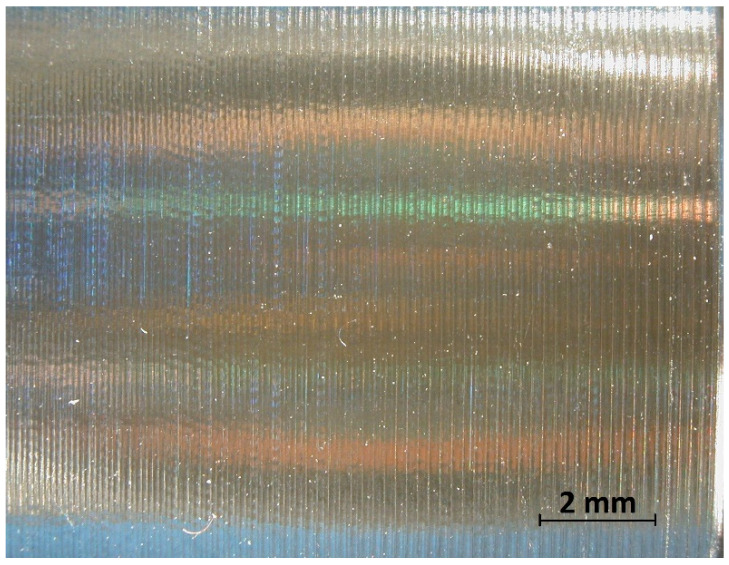
View of the machined surface after taper 45° turning without coolant (36 HRC).

In the analysis of the Δ*Rz* coefficient, it was found that the influence of turning dynamics on the machined surface quality was great. Turning of material with a hardness of 23 HRC with the use of a coolant allowed us to obtain a value for that coefficient close to 1 or even below 1. This meant that, with the use of coolant, the foreseen parameter *Rz_to_* was close to the values obtained during investigation. The situation was similar for turning with a straight-line trajectory (Figure 12) and for turning with a curved trajectory (Figure 13). It should be pointed out that the material with a hardness of 23 HRC at a cutting speed of *v_c_* = 70 m/min with cooling has always resulted in a Δ*Rz* coefficient below 1. Turning of the material with a hardness of 36 HRC resulted in an increase in the coefficient under consideration, as compared to each considered case in relation to the material with a hardness of 23 HRC. The similarity to the Δ*Rz* coefficient for the material with a 23 HRC hardness was that the lowest value of the coefficient was obtained when turning with a linear trajectory of motion for the cutting speed *v_c_* = 70 m/min with the use of coolant (Figure 14). In most cases under analysis, the Δ*Rz* coefficient was far above 1. The maximum value of the Δ*Rz* coefficient was recorded during turning with a curved trajectory at a cutting speed of *v_c_* = 45 m/min without cooling (Figure 15). In that case, the value of Δ*Rz* = 3.393 µm. This showed a very strong instability of the process. The reason for such a great difference between the actual *Rz* value and the foreseen one, *Rz_to_*, was vibration and stickings on the machined surface during machining without cooling (Figure 16 and Figure 17).

**Figure 18:**
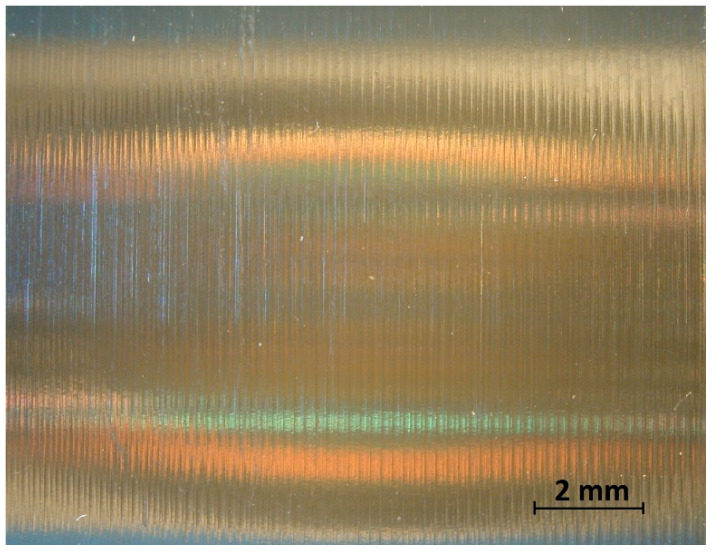
View of the machined surface after longitudinal turning without coolant (23 HRC).

**Figure 19:**
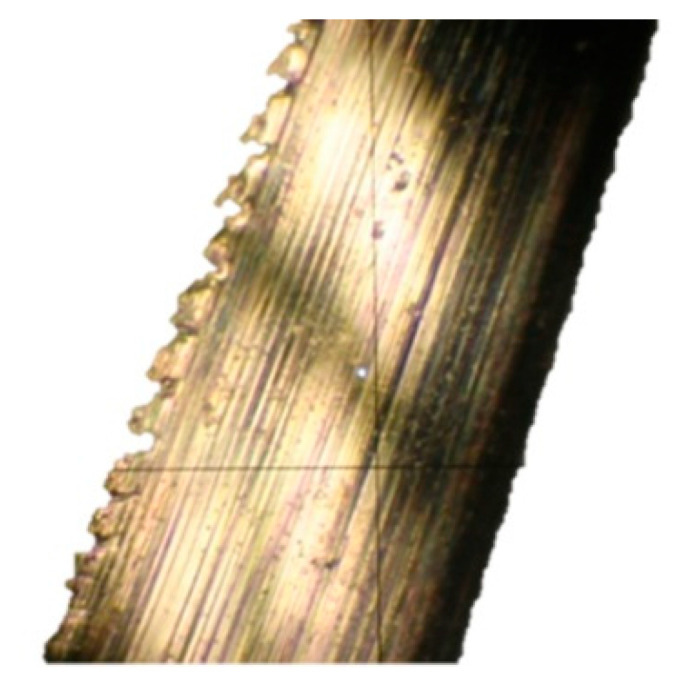
View of the chip shape after longitudinal turning without coolant (36 HRC).

In machining of material with a hardness of 23 HRC, vibration was not recorded, but stickings appeared on the machined surface (Figure 18). The stickings on the machined surface most likely originated from the generated chips or, more strictly, from the fuzzed edge that arose at the location of the smallest thickness of the cut material (Figure 19).

**Figure 20:**
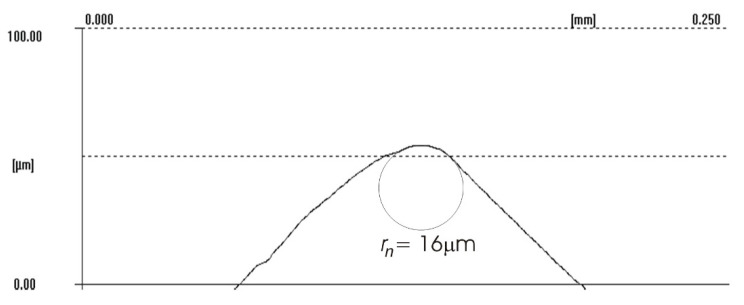
Cutting edge radius *r_n_* of the new cutting insert.

**Figure 21:**
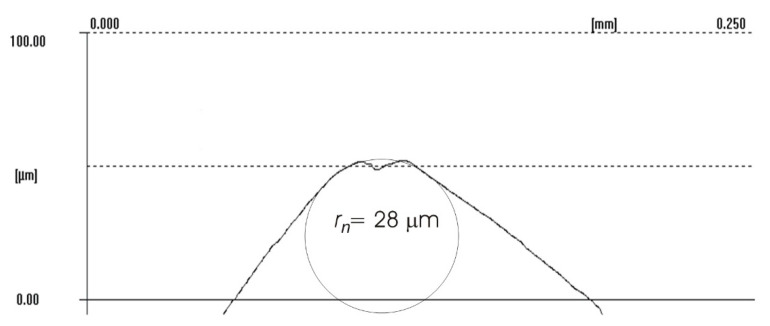
Cutting edge radius *r_n_* of the worn cutting insert after longitudinal turning with coolant (23 HRC).

**Figure 22:**
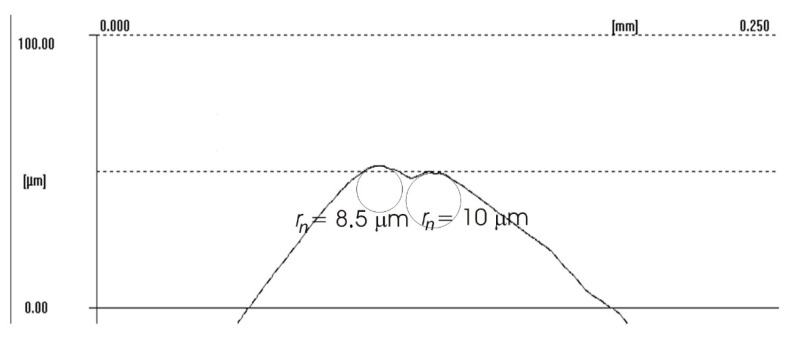
Cutting edge radius *r_n_* of the worn cutting insert after longitudinal turning with coolant (36 HRC).

**Figure 23:**
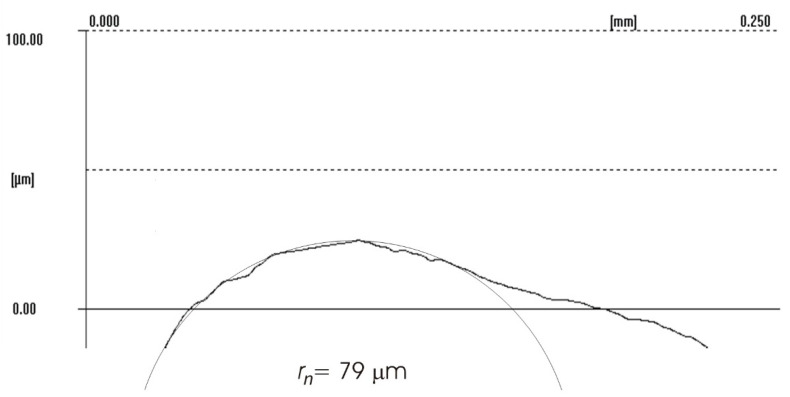
Cutting edge radius *r_n_* of the worn cutting insert after longitudinal turning without coolant (23 HRC).

**Figure 24:**
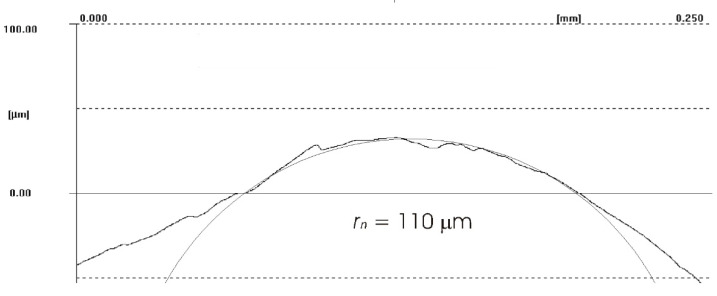
Cutting edge radius *r_n_* of the worn cutting insert after longitudinal turning without coolant (36 HRC).

The difference between the considered *Rz* parameter and the theoretical *Rz_to_* value resulted from a number of factors. One of them was the applied coolant, which, in addition to leading heat out of the cutting zone, also created a lubricating film between the cutting edge and the material under machining. The coolant also washed chip fragments, preventing their sticking to the machined surface. Formation of singeing on the machined surface was not observed in any of the considered cases. Another cause of the difference between the actual *Rz* parameter value and the theoretical one was the change in the cutting edge radius, *r_n_*, during machining. Prior to machining, the edge had a radius of *r_n_* =16 µm (Figure 20). The measured value of that radius after machining was, to a large extent, dependent on the conditions of machining. Turning with the use of coolant caused a sort of “withdrawal” of the cutting edge (Figure 21 and Figure 22). The symptoms of wear were similar for both examined hardnesses of the machined material; however, for the higher hardness, the withdrawal of the cutting edge was a bit larger. Much greater wear of the cutting edge was observed in dry machining. Rounding of the cutting edge in longitudinal turning of the 23 HRC material was almost five times larger than on a new edge (Figure 23). In the case of machining the 36 HRC material, rounding of the cutting edge was still larger, almost seven times that of a new edge (Figure 24). Such a large increase of the cutting edge rounding radius, *r_n_*, directly influenced the value of the minimum uncut chip thickness value, *h_min_*, and increased the surface of the cutting edge’s contact with the machined material. The larger contact surface generated more friction, which generated more heat in the cutting zone. One of the properties of nickel alloys is a low coefficient of heat conduction [38,39], which results in the cutting edge leading out more heat than chips and the machined material, as is the case when machining steel.

**Figure 25:**
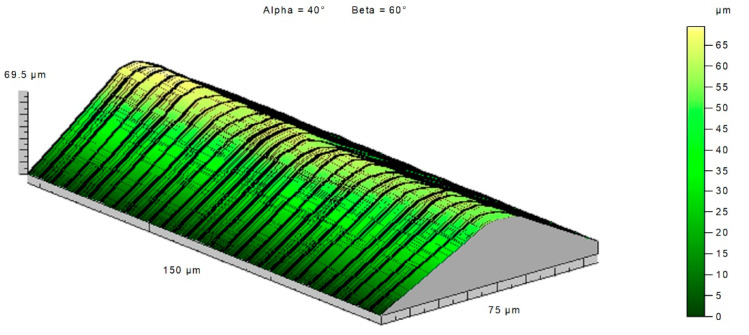
Topography of the cutting edge of a new insert.

**Figure 26:**
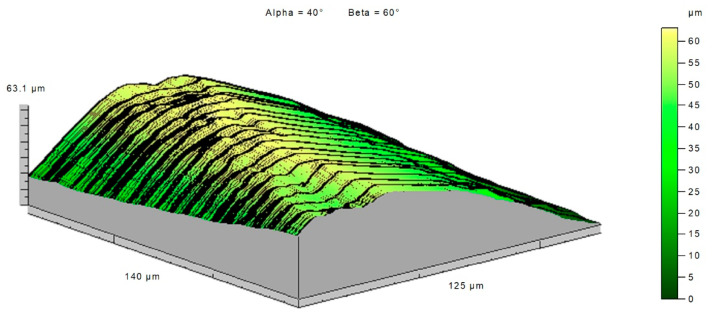
Topography of the cutting edge of a worn insert.

The topography of the cutting edge before machining (Figure 25) and after machining (Figure 26) showed how great changes took place on the cutting edge. The topography of a fragment of the plate used in the tests showed that the changes that took place on the cutting edge were even in individual cross sections. The value of the wear most likely was influenced by the variable section of the cut layer along the cutting edge.

**Figure 27:**
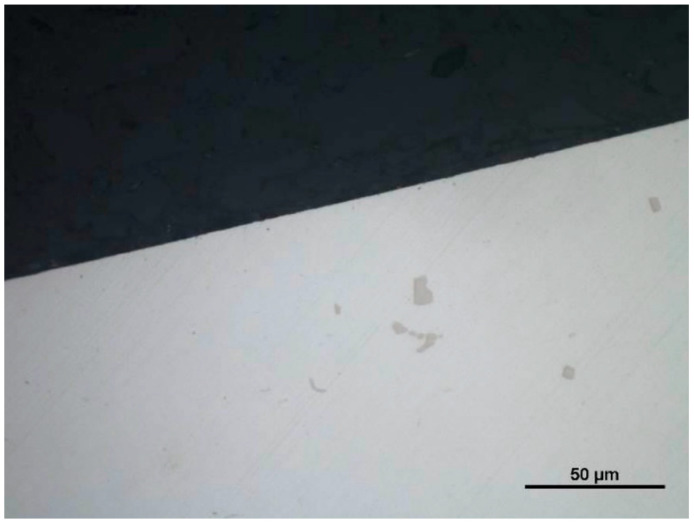
A view of a microsection after longitudinal turning with cooling and before etching.

**Figure 28:**
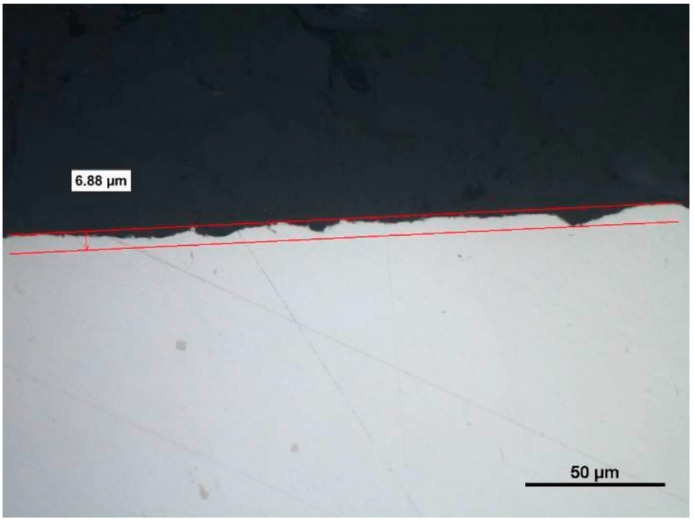
A view of a microsection after longitudinal turning without cooling and before etching.

Metallographic examination confirmed the anomalies with significant differences from the theoretical values of the surface machined without the use of coolant (Figure 27 and Figure 28). A view of a microsection after longitudinal turning without cooling showed many disturbances that influenced the values of *Ra* and *Rz* parameters. The average values of the roughness parameters for machining the 23 HRC material with the use of coolant, for a cutting speed of *vc* = 70 m/min, were *Ra* = 0.887 µm and *Rz* = 3.842 µm. No use of coolant resulted in an increase of those parameters up to the values of *Ra* = 1.580 µm and *Rz* = 6.497 µm. Comparison of the two examined cases proved drastic deterioration of the quality of the machined surface: the *Ra* parameter grew by almost 80%, while the *Rz* parameter grew by 70%.

**Figure 29:**
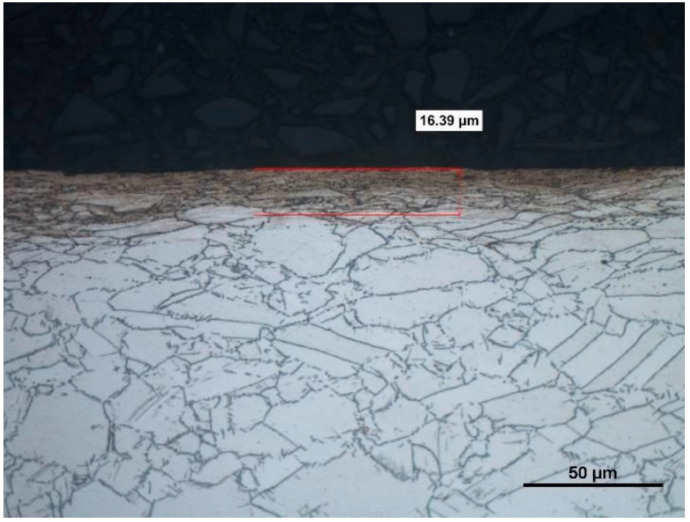
A view of a microsection after longitudinal turning without cooling and after etching.

**Figure 30:**
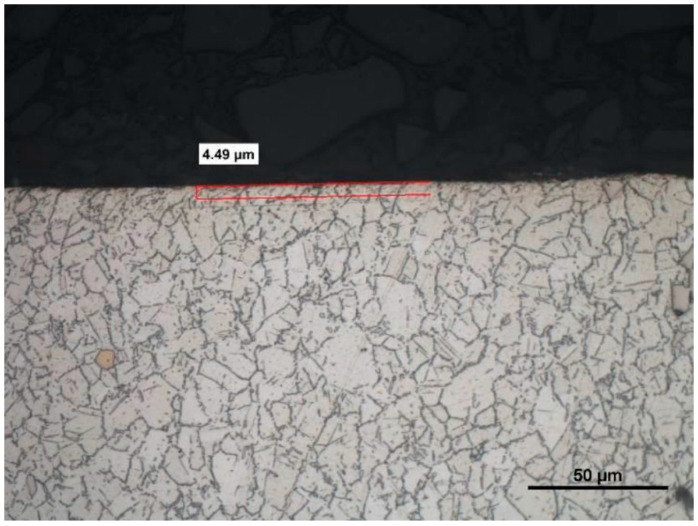
A view of a microsection after longitudinal turning with cooling and after etching.

Analysis of the microstructure proved that during machining without coolant, the generated heat penetrated the material under machining, changing the microstructure in its top layer (Figure 29). Ezugwu et al. [20] observed that the properties of Inconel 718 resulted in the deformation of the cutting edge and the part under machining, which was the reason for generation of heat and plastic deformations. Generated heat usually changes the microstructure of the alloy and causes stresses arising in the top layer. The temperature and deformation, together or separately, can cause cracks and microstructure changes during machining. The influence of heat on the change of microstructure was not observed in machining with the use of coolant (Figure 30). The influence of the cutting insert on deformation of grains was visible. The depth of microstructure changes for the case under consideration was 4.49 µm; for dry machining, the changes reached over 16 µm (i.e., almost four times as much).

## 4. Conclusions

Basing on our own experimental investigation, we formulated the following conclusions:

- It was found that a higher cutting speed, with the other machining conditions being kept the same, allowed us to obtain a better surface quality as assessed by the roughness parameters, *Ra* and *Rz*. The least average values of those parameters, for a cutting speed of *v_c_* = 45 m/min, were: *Ra* = 1.233 µm and *Rz* = 5.154 µm for the 23 HRC material; and *Ra* = 1.475 µm, *Rz* = 6.592 µm for the 36 HRC material. For a cutting speed of *v_c_* = 70 m/min, the least average roughness parameters were *Ra* = 0.843 µm and *Rz* = 3.697 µm for the 23 HRC material
- Dry turning of the Inconel 718 alloy, regardless of its hardness, influenced the character and size of the wear of the cutting edge. No use of coolant resulted in an increase in the radius of the cutting edge rounding, and consequently, an increase in the amount of heat generated in the cutting zone, a symptom of which was changes in microstructure and quality deterioration of the machined surface.
- The investigation results proved the influence of the angle between the cutting edge and the machined surface on the roughness parameters, *Ra* and *Rz*. Reduction in that angle caused an increase in the active length of the cutting edge, as a result of which higher cutting resistances were generated, manifested by vibrations that negatively influenced the surface roughness.
- The accuracy of the application of Formula (5) to predict the value of parameter *Rz_to_* was sufficient in stable conditions of the machining process. Disturbance of that stability by the lack of coolant application, increase of the active length of the cutting edge, and change of the cutting speed influenced the dynamics of the machining process; i.e., the value of Δ*Rz_d_*. Analysis of the Δ*Rz* coefficient indicated that the largest influence on value of Δ*Rz* was that of the lack of coolant application in turning, and consequently, the lack of lubrication between the cutting insert and the machined surface. The Δ*Rz* coefficient analysis also showed that the cutting speed had a significant influence on the surface roughness when cutting the Inconel 718 alloy. The use of a cutting speed of 70 m/min during cooling turning allowed us to obtain a Δ*Rz* value below 1.
- The process of dry turning of the Inconel 718 alloy resulted in the formation of stickings on the machined surface. This phenomenon was not observed in machining with cooling. Therefore, the material could not be dry machined in the process of finish turning.

## Data Availability

Data sharing is not applicable to this article.
